# Uterine Incarceration: Rare Cause of Urinary Retention in Healthy Pregnant Patients

**DOI:** 10.5811/westjem.2015.7.27185

**Published:** 2015-10-20

**Authors:** Richard Slama, Mike Barry, Ken McManus, Doug Latham, Matthew Berniard

**Affiliations:** Naval Medical Center Portsmouth, Department of Emergency Medicine, Portsmouth, Virginia

## Abstract

Gravid uterine incarceration (GUI) is a condition that is well discussed in literature; however, there are few acute diagnoses in the emergency department (ED). We present a case series where three multiparous females presented to the ED with non-specific urinary symptoms. On bedside ultrasound, each patient was noted to have a retroverted uterus and inferior bladder entrapment under the sacral promontory. GUI is a rare condition that can lead to uremia, sepsis, peritonitis, and ultimately maternal death. Emergency physicians should include GUI in their differential diagnosis in this patient population and use bedside ultrasound as an adjunct to diagnosis.

## INTRODUCTION

Gravid uterine incarceration (GUI) is a relatively rare condition that results in the uterus becoming trapped between the sacral promontory and the pubic symphysis during pregnancy.^1^ As the uterus becomes more gravid, the cervix becomes superiorly displaced and can eventually lead to bladder outlet obstruction. We report a case series of uterine incarceration where otherwise-healthy patients presented to the emergency department (ED) between approximately 13 weeks and 21 weeks estimated gestational age with dysuria, urgency, frequency, and low back pain after being recently seen by obstetrics and gynecology (OBGYN) which could not determine the cause for the patients’ symptoms. In this case series we present three cases of uterine incarceration diagnosed by ultrasound in the ED, discuss previously published cases, and discuss the implications for emergency physicians.

## CASE REPORT

Three multiparous patients with pertinent history and symptomatology (Table) presented to the ED with dysuria, urgency, frequency, and low back pain between two and six weeks in duration. Further review of systems was otherwise negative. The patients denied all toxic habits including alcohol, drugs and tobacco, and did not use any medications other than prenatal vitamins.

Patient #1 had been seen recently by OBGYN where she underwent evaluation for dysuria/urinary tract infection that was negative. Because of her unremitting symptoms and despite reassurance, she came to the ED for evaluation. Initial laboratory evaluation of the patient revealed only bacterial vaginosis. A bedside ultrasound assessment was performed that showed normal-appearing kidneys, and was negative for free abdominal fluid or pericardial effusion. The inferior bladder pole was entrapped by the gravid uterus, and contained a significant volume of urine (Figure).

Upon these findings, the patients underwent straight Foley catheterization with return of 180mL urine that resulted in alleviation of symptoms.

Patient #2 presented with progressively increasing difficulty with urination over a period of two weeks. She underwent physical exam and formal laboratory evaluation that was unrevealing for any infectious process. She was evaluated by bedside ultrasound, which again revealed trapping of the bladder pole by the gravid uterus. She also underwent straight catheterization and was instructed on self-catheterization with next-day follow up with OBGYN.

Patient #3 had a more acute onset of her retention that developed over a two-day period. She was evaluated in a similar manner to patients #1 and #2 with the only abnormal finding being bacterial vaginosis. Her bedside ultrasound showed a retroflexed uterus and a significant amount of urine in the bladder. Because of her symptoms she was sent for a formal pelvic ultrasound in the ED, which confirmed the diagnosis of uterine incarceration and showed >600mL in the bladder. She underwent straight catheterization with return of 400mL urine.

All three patients received formal ultrasounds confirming compression of the inferior pole of the bladder. OBGYN was consulted and examined the patients in the ED, ultimately deciding to discharge the patients with close follow up the next day. The patients were given instruction on self-catheterization, and return precautions should their condition worsen.

They were seen the next day in the OBGYN clinic and underwent a trial of pessary placement with successful alleviation of their symptoms. Three weeks later the patients’ symptoms had improved to the point where they no longer required use of the pessary.

## DISCUSSION

The exact mechanism of GUI is believed to be due to trapping of the uterine fundus in a retroverted position, which leads to a progressively elongated cervix that becomes displaced anteriorly and leads to obstructive bladder symptoms.^2^ Risk factors for this condition include post-surgical adhesions, pelvic inflammatory disease, fibroids, and laxity of supporting tissues.^3^ The most typical presentation occurs between 14 and 16 weeks of gestation with a variety of symptoms mimicking common gastrointestinal, genitourinary, and musculoskeletal conditions. Physical findings include anterior displacement of the uterus, anterior angulation of the vaginal angle, retroverted uterus, cervical displacement toward cephalad and a low-lying fundal height for gestational age.^4^

Though urinary tract infections (UTI) are by far the most common cause of dysuria in pregnant patients, a patient with a GUI can easily be misdiagnosed as a UTI even by experienced clinicians. Though these patients’ particular presentations did not appear alarming, they could have easily been disregarded as normal pregnancy pain or Braxton-Hicks contractions if careful attention to detail was not made. The complications of a missed GUI are rare, but could be potentially disastrous and life threatening. These complications include hydronephrosis, UTI, bladder rupture, sepsis, peritonitis, miscarriage, oligohydramnios, fetal growth restriction, and fetal demise.^5^–^7^ Even if these immediate complications are not present, delayed complications can include a pregnancy loss of up to 33% in the second trimester.^8^,^9^ Because of the potential of these serious complications, this is a diagnosis that should be considered more frequently in the ED, especially in community care settings such as ours, where obstetric patients make up a large portion of the ED census per year.

Though GUI has been extensively described in the literature, there are few reports of its actual diagnosis in the ED setting. During a literature search we did find one case report where an ED noted that a patient had a clinically incarcerated uterus; however, there was no ultrasonographic evidence of bladder obstruction in this particular case.^10^ We believe that our case series is the first known series of GUI diagnosis in the ED using bedside ultrasonography. Although there are no established gold standard tests for GUI, both ultrasound and magnetic resonance imaging seem to be acceptable modalities for confirming the diagnosis.^11^,^12^ This case demonstrates one of the many utilities of ultrasound in the ED setting, particularly in experienced operators. While no conclusions about statistical significance of testing for GUI can be drawn from this particular test, we emphasize two main points from our experience.

The first is that GUI is a rare, potentially fatal, but possible diagnosis in all pregnant women with symptoms of UTI and/or bladder obstruction that should be in the differential diagnosis for emergency physicians. Second, we believe that when used in conjunction with clinical findings, bedside emergency ultrasound is an excellent adjunct to aid in the diagnosis of GUI.

## Figures and Tables

**Figure:**
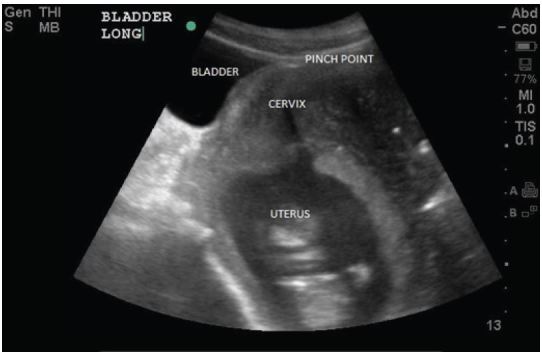
Inferior bladder, containing significant volume of urine, entrapped by gravid uterus.

**Table:** Multiparous patients with symptoms indicating possible gravid uterine incarceration.

Patient	Age	EGA	Parity	PMH/comorbidities	Symptoms	Outcome
1	37	13	G6P2122	None	Urinary retention, bilateral costo-vertebral tenderness and thick white discharge on speculum exam	Self-catheterization, Pessary
2	42	13	G5P3013	Infertility, LEEP, salpingectomy	Urinary retention	Self-catheterization, pessary placement
3	22	21	G15P5009	Multiple spontaneous abortions, clotting disorder	Urinary retention, LUQ pain, nausea	Pessary placement

*EGA*, estimated gestational age; *PMH*, past medical history; *LEEP*, look electrosurgical excision procedure; *LUQ*, left upper quadrant
